# Application of BCXZM Composite for Arsenic Removal: EPS Production, Biotransformation and Immobilization of *Bacillus* XZM on Corn Cobs Biochar

**DOI:** 10.3390/biology12040611

**Published:** 2023-04-18

**Authors:** Sana Irshad, Zuoming Xie, Mao Qing, Asad Nawaz, Sajid Mehmood, Suliman Yousef Alomar, Muhammad Faheem, Noman Walayat

**Affiliations:** 1 School of Civil and Transportation Engineering, Shenzhen University, Shenzhen 518000, China; 2 School of Environmental Studies, China University of Geosciences, Wuhan 430074, China; 3 Institute for Advanced Studies, Shenzhen University, Shenzhen 518000, China; 4 State Key Laboratory of Biogeology and Environmental Geology, China University of Geosciences, Wuhan 430074, China; 5 Key Laboratory of Agro-Forestry Environmental Processes and Ecological Regulation of Hainan Province, Hainan University, Haikou 570228, China; 6 Zoology Department, College of Science, King Saud University, Riyadh 11451, Saudi Arabia; 7 Centro Tecnológico de la Carne de Galicia, Parque Tecnológico de Galicia, San Cibrán das Viñas, Rúa Galicia N 4, 32900 Ourense, Spain

**Keywords:** *Bacillus* XZM arsenic, bio-removal/bioremediation, multifactorial biochar, adsorption, chemo-metric models, desorption

## Abstract

**Simple Summary:**

Biological techniques are environmentally friendly for arsenic bioremediation; however, the practical applicability for these techniques requires some engineering additions. For instance, in the bacterial bioremediation of arsenic, after the bioaccumulation of arsenic, the bacterial biomass is easily suspended in the water and difficult to separate. To resolve this problem, the current research provides a simple approach. Arsenic-tolerant bacterium *Bacillus* XZM was immobilized on Biocahr and biofilm provided a filter layer for arsenic adsorption. This composite was able to bio-sorb and bio-accumulate 42.3 mg/g of arsenic, which implies that it required USD 6.24 for the bioremediation of 1000 gallons of drinking water (with 50 µg/L of arsenic). This research is beneficial and worthy as it provides a technological application of a biological technique for arsenic-free drinking water. Moreover, it is a clean, green and economical method as it generates no residual sludge and does not use any harmful chemicals.

**Abstract:**

This study determined the effect of *Bacillus* XZM extracellular polymeric substances (EPS) production on the arsenic adsorption capacity of the Biochar-*Bacillus* XZM (BCXZM) composite. The *Bacillus* XZM was immobilized on corn cobs multifunction biochar to generate the BCXZM composite. The arsenic adsorption capacity of BCXZM composite was optimized at different pHs and As(V) concentrations using a central composite design (CCD)2^2^ and maximum adsorption capacity (42.3 mg/g) was attained at pH 6.9 and 48.9 mg/L As(V) dose. The BCXZM composite showed a higher arsenic adsorption than biochar alone, which was further confirmed through scanning electron microscopy (SEM) micrographs, EXD graph and elemental overlay as well. The bacterial EPS production was sensitive to the pH, which caused a major shift in the –NH, –OH, –CH, –C=O, –C–N, –SH, –COO and aromatic/-NO_2_ peaks of FTIR spectra. Regarding the techno economic analysis, it was revealed that USD 6.24 are required to prepare the BCXZM composite to treat 1000 gallons of drinking water (with 50 µg/L of arsenic). Our findings provide insights (such as adsorbent dose, optimum operating temperature and reaction time, and pollution load) for the potential application of the BCXZM composite as bedding material in fixed-bed bioreactors for the bioremediation of arsenic-contaminated water in future.

## 1. Introduction

Toxic metal contamination, particularly arsenic, has become a major concern in the modern world [1]. Arsenic poisoning exists all over the globe and it is estimated that >170 M people all over the world are facing an arsenic-contaminated environment. The condition becomes worse when it affects populous regions of the world, such as in Southeast Asia, where ~100 M people are at risk of arsenic poisoning [2]. Although arsenic is a micronutrient for many species, excess exposure to arsenic is has harmful health effects [3].

The arsenic concentration in uncontaminated water is <1 or 2 µg/L. This concentration has increased up to 500 times higher than that listed in drinking water guidelines (10 μg/L) due to volcanism, the weathering of arsenic-containing sediments and industrialization [4]. As a result, arsenic poisoning is causing many diseases in humans, such as skin lesions, digestive track poisoning, liver and kidney disorders, brain cancer melanosis, leucomelanosis, dorsum, keratosis, hyperkeratosis, non-petting skin cancer and oedema [5]. The remediation of arsenic, especially from contaminated drinking water, is a priority as it is directly connected to the food chain. Arsenic pollution is complex to remediate as compared to other heavy metals, as arsenic exists in the form of oxyanions rather than cations [2]. Arsenic bioremediation [6,7,8,9] is more environmentally friendly than physical and chemical methods (such as coagulation, flocculation, and membrane filtration) that are being used for the remediation of arsenic-contaminated water [10].

Among the bioremediation techniques, bacteria play a vital role in the mobilization, transformation and bioremediation of arsenic through complex metabolic processes of reduction, oxidation, methylation and volatilization. Many of the bacterial species, e.g., *Bacillus* sp. [11], *Bacillus flexus* and *Acinetobacter junii* [12], *Pseudomonas* sp. [13], and *Achromobacter* sp. [7], have been used for arsenic bioremediation in many laboratory experiments as bacteria can grow as biofilm or attach to a surface in the form of colonies [14]. A surface is a key requirement for the formation of biofilm by bacteria. However, for arsenic removal, the microbes/biomass poses certain issues regarding solid–liquid separation, swelling and special work conditions. Therefore, it is practically feasible to immobilize the free biomass on a surface for a higher removal efficiency [15].

The reutilization of waste biomass can be an effective substrate for biofilm immobilization [16,17]. The key is to use biomass in a form that can be easily regenerated for reuse, which should be deliberated in the application of simultaneous biosorption and bioaccumulation (SBB) for the elimination of toxic metals from natural water or wastewater. Recently, immobilization bacteria have been reported on neem leaves/MnFe_2_O_4_ composite [18], rice straw [19] and sawdust/MnFe_2_O_4_ composites [20] for the bio-removal of arsenic, in both batch and fixed-bed reactors. However, there are some issues related to the utilization of these materials that should be addressed for the application of these materials, e.g., sawdust is biodegradable and once bacteria have grown, it is biodegraded and cannot be recycled. Similarly, neem leaves are well-proven to have anti-bacterial properties; therefore, not all kinds of bacteria can grow on this material to make biofilm. As an alternative, biochar can act as an ideal substance for the immobilization of bacteria owing to its low degradation rate. Another advantage of biochar is its porous structure that is responsible for the high surface area. Therefore, due to porous surface and being a comparatively inert material, biochar can provide a large space for the growth of biofilm [21]. In addition to this, SBB is a very different process compared to the simple biosorption of arsenic using biosorbents without biofilms, which are based on kinetic linear expressions [18]. The simple kinetic linear expressions, especially the Langmuir and Freundlich isothermal models, cannot be applied on this process, as these are linear models and involve a limited number of factors (mostly two). These are not suitable in the case of SBB that is directly dependent on the multifactorial bacterial growth for the arsenic adsorption. The removal of As(V) through SBB is found to be filled with Brouers–Sotolongo and Fritz–Schlunder-V isotherm models while using *Corynebacterium glutamicum* immobilized on NL/MnFe_2_O_4_ composite for the removal of arsenic [22]. However, these are non-linear models and not very simple to apply, similarly to the Langmuir and Freundlich models [23]. Secondly, the bacteria used in this process have a major role in the arsenic removal. Undoubtedly, the process as a synergistic effect of bacteria and biosorbent is found to be more effective than a simple biosorption process; however, the individual role of bacteria in terms of arsenic redox transformations and metabolisation is not studied very clearly [19,24]. Moreover, the maximum adsorption capacity of a biosorbent-bacteria composite at specific operation conditions remains undetermined [25].

Multivariate models can offer a solution for the optimization of SBB as it can simultaneously evaluate many factors with the effective monitoring of the interaction between process parameters. The experimental design can be formulated for the prediction of maximum removal/bioremediation efficiency using simulation techniques [26,27]. Response surface methodology (RSM), especially central composite design CCD, can be applied to multifactorial processes such as SBB to attain optimum conditions for adsorption of arsenic. In addition to this, CCD/RSM is less laborious than isotherm models. It can simultaneously promote the assessment of many parameters without any information loss, consequently improving the effectiveness of the process [27,28].

Therefore, the current study is an environmentally friendly approach that used corn cobs biochar for As(V) bioremediation, which is an approach for agricultural waste management. The specific aims of the current study are: (1) the immobilization of indigenous strain *Bacillus* XZM on corn cobs multifunction biochar to develop a Biochar-*Bacillus* XZM (BCXZM) composite for the bio-removal of As(V) from contaminated water at various pH and arsenic concentrations; (2) the introduction of the multivariate RSM/CCD model for the determination of the maximum arsenic adsorption capacity of BCXZM composite; (3) the evaluation of the role and importance of the *Bacillus* XZM in the SBB process; and (4) the evaluation of the cost of BCXZM composite preparation.

## 2. Methodology

### 2.1. Bacterial Isolation and Identification

The arsenic-transforming and EPS-producing indigenous strain *Bacillus* XZM (SUB4899103 BacillusMK271736) was isolated from the deep ground water of Shuangzhai village Dotong Basin, Shanxi, China (39°21′ N, 112°51′ S), as a part of our previously reported work. It can tolerate high arsenic concentrations and carryout redox transformations, which are required for arsenic bioremediation [29]. The strain was acclimatized to 50 ppm As(V) in nutrient broth (NB) media (0.5% peptone, 0.2% yeast extract and 0.5% NaCl) at 32 °C overnight prior to BCXZM preparation.

### 2.2. Determination of Extracellular Polymeric Substance (EPS) Production by Bacillus XZM

The EPS production by *Bacillus* XZM was quantified according to Kazy, Sar, Singh, Sen and D’souza [9] at various As(V) concentrations (0 to 80 ppm) and pH (3 to 9 at 50 mg/L As(V), separately. Briefly, the bacterium XZM was incubated in liquid media (yeast extract 10 g/L, MgSO_4_·7H_2_O 0.14 g, (NH_4_)_2_SO_4_ 0.3 g/L, CaCl_2_·2H_2_O 0.2 g/L, NaCl 0.1 g/L, H_3_BO_3_ 0.6 mg/L, glucose 10 g/L and sucrose 50 g/L) at 130 rpm and 32 °C. As shown in Figure S1, most EPS can be formed at 32 °C once different temperatures between 25 and 40 °C are tested. Thus, an optimum temperature 32 °C was selected for the experimentation in this study. The growth was continued for 72 h and bacterial cells were harvested through centrifugation at 15,000× *g* for 30 min. The supernatant was doubled in volume with ice cooled ethanol (95%) and kept static for 24 h at 4 °C. The precipitates of EPS formed were collected through centrifugation at 18,000× *g* for 30 min at 4 °C and cleaned thrice with ethanol. The dry weight of collected precipitates was measured as the amount of EPS produced.

### 2.3. Preparation of BCXZM Composite

The multifunction biochar was prepared from corn cobs according to Bao, et al. [30] and the BCXZM composite was prepared via the immobilization of *Bacillus* XZM on biochar according to the previously defined method of Podder and Majumder [31] with a slight modification. Briefly, approximately 5 mL of bacterial culture was inculcated in 495 mL of NB media and incubated at 32 °C for 24 h. After this, 0.4 g of the biochar (0.8 g/L) was added in this bacterial suspension and placed in the shaking incubator (120 rpm) at 32 °C for 24 h and filtered afterward. Twenty-two bottles were incubated at the same time and the BCXZM composite was collected and immediately used for the arsenic adsorption optimization experiment. All the processes of inoculation, addition and filtration were performed in a sterilized and anaerobic chamber.

### 2.4. Multivariate Optimization

Regarding adsorption, multivariate optimization was performed using response surface methodology, and central composite design (CCD)2^2^ using Design Expert 11 software. The two factors pH and As(V) concentration were adjusted at high and low levels. The pH was adjusted between 5.5 and 7, while As(V) concentration ranged from 15 to 45 ppm (Table S1). The treatments obtained are shown in Table 1. After adding all the responses, the conditions for maximized/optimized arsenic adsorption were also simulated via model application.

### 2.5. Experimental Set-Up

The adsorption experiment was performed in NB media. The Na_2_HAsO_4_ was used as a source of arsenate stress, while 1 N HCl and NaOH was used for pH adjustments. Before the establishment of the multivariate experimental set-up, the optimum dose and contact time of BCXZM for As(V) removal were determined. For the optimum dose determination, ten different doses (0.5 to 5 g/L) of the BCXZM composite were added in the CDM containing 50 mg/L of As(V) and placed overnight in the shaking incubator at 32 °C. As shown in Figure S2a, 2 g/L was selected for the optimum dose. For the optimum contact time determination, 2 g/L BCXZM composite was added in the CDM with 50 mg/L of As(V) and 1 mL the sample was collected after every hour, filtered (1 µm) and stored after preservation (0.5% HNO_3_) at −20 °C before arsenic determination (same method of sample storage was followed for the whole experiment). As a result, 6 h was selected as optimum contact time (Figure S2b). The adsorption experiment for 11 treatments of (CCD)2^2^ optimization was conducted for 6 h and, during this time period, the samples were incubated in an orbital shaker (120 rpm) at 32 °C, while the BCXZM composite dose was 2 g/L. All the experiments were conducted in duplicates; in this way, 22 microcosms were established in air-tight glass bottles with 100 mL of CDM for each. After this, the samples were removed from the shaking incubator and carefully filtered (Wattman filter # 42) to obtain BCXZM and filtrate for further analysis. The filtrate was further filtered with 1 µm filters and stored at −20°C, after preservation (0.5% HNO_3_), for arsenic determination. The arsenic adsorption (*q*) was calculated according to Equation (1), and the values were computed in the RSM model to determine the optimized value of pH and As(V) to attain maximum arsenic adsorption by BCXZM composite.

(1) $$\;q=\frac{C_{o}-C_{eq}\;}{m}V$$

The *q* is measured in mg/g. The *C_o_* and *C_eq_* are the initial and equilibrium concentrations of arsenic (mg/L), while *m* and *V* show the mass of adsorbent (g) and volume of the solution (mL).

The same process of arsenic adsorption experiment was followed for the model simulated solution to attain maximum arsenic adsorption. For the purpose of comparison, the same experiment was also conducted for biochar as the adsorbent in optimized conditions. Furthermore, all inoculation and filtration process were performed in an anaerobic chamber under completely sterilized conditions. The liquid samples were filtrated (1 µm), preserved (0.5% HNO_3_) and stored at −20 °C in the dark for further arsenic determination using hybrid generation-atomic fluorescence spectroscopy (HG-AFS; AFS-830, Beijing Jitian Instrument Co., Ltd., Beijing, China). The BCXZM composite was pretreated for further analysis as described in Section 2.6, Section 2.7 and Section 2.8.

### 2.6. FTIR Spectroscopy

The structural variations in the BCXZM composite before and after adsorption of arsenic were studied using an FT-IR spectrometer (Nicolet 380, Thermos Scientific Inc., Waltham, MA, USA) at room temperature. The samples were pressed with KBr to make pallets after freeze-drying and scanning was carried out 64 times from 400 to 4000 cm^−1^ at a resolution of 4 cm^−1^. The spectra were recorded after the elimination of background interferences and analyzed using OMNIC professional Software version 8.3.

### 2.7. Scanning Electron Microscopy (SEM) and Energy Dispersive X-ray Spectroscopy

The BCXZM composite was carefully collected via filtration and fixed in 3% glutaraldehyde for 6 h after being washed with deionized water. After fixation, the BCXZM samples were washed with serially diluted ethanol (40 to 100%), air-dried in a sterilized chamber and observed under a scanning electron microscope (Model: JSM-6390LV; NTC, Tokyo, Japan). The BCXZM composite and biochar after the adsorption in optimized conditions were also analyzed for EDX graph and elemental overlay of arsenic using SEM (JSM-6700F NTC, Tokyo, Japan) fitted with an energy-dispersive X-ray spectroscopy set-up at 15 kV (penetration depth of the electrons: ∼2.2 µm).

### 2.8. Arsenic Desorption Assay

A desorption study was performed for the optimized experiment resulted after multivariate analysis. Approximately 0.3 g of the BCXZM composite, after adsorption of arsenic, was added into 100 mL of NaOH solution at a concentration of 0, 0.01, 0.05, 0.1, 0.5, and 1 M, separately. The mixtures were then placed into an orbital shaker at 32 °C, 120 rpm for 110 min. The amount of arsenic was accessed in the residual solution and the percentage of desorbed arsenic was determined as in Equation (2):

(2) $$Desorption\left(\%\right)=\frac{Released\;arsenic\;\left(ppm\right)}{intially\;adsorbed\;arsenic\;\left(ppm\right)}\times 100$$

### 2.9. Cost of Making BCXZM Composite

The cost of BCXZM preparation included the cost of nutrients, water, biochar and electricity. The cost of biochar production was considered to be the same as previously given for the biochar preparation from agricultural residue [32]. The nutrient chemicals and water price corresponded to those of vendor quotes/standard engineering estimates.

### 2.10. Statistical Analysis

The experiment was performed in duplicates to calculate mean and standard deviation. The mean and standard deviation (Mean ± S.D) were executed using SPSS 19.0 for Window 10 (SPSS Inc., Chicago, IL, USA).

## 3. Results and Discussion

### 3.1. Bacterial Growth, EPS Production and As(V) Reducing Efficiency

**Figure 1:**
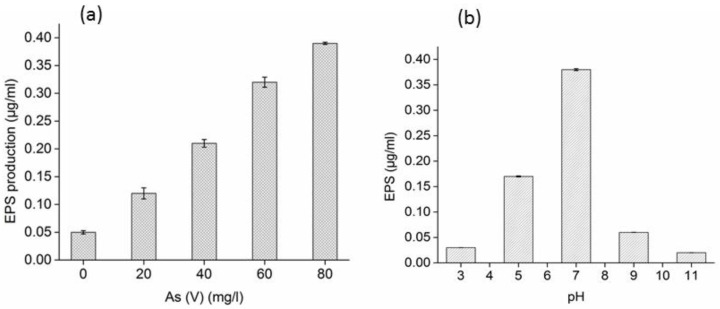
(**a**) EPS production by *Bacillus* XZM at different at arsenic concentrations and (**b**) pH. Note: Mean ± Standard deviation (n = 2); in (**b**), As(V) level of 50 mg/L was selected based on (**a**).

The EPS production by strain XZM is shown in Figure 1. The EPS production was increased with the increasing arsenic concentration; an increase of roughly two folds in EPS was observed by doubling the arsenic concentration (Figure 1a). At 50 mg/L As(V), the EPS production was found to be maximum at pH 7, while it was strongly reduced at acidic and basic pH values (Figure 1b). This can be justified mainly in terms of strain XZM sensitivity for pH fluctuations. Many studies in the past have reported the increase in EPS production by arsenic-resistant bacteria with increasing As(V) concentration [6,15,24,33]; similarly, the reduction in EPS production under the influence of pH was also confirmed by Kang, et al. [34] of bacterial communities.

### 3.2. Effect of As(V) Concentration and pH on Arsenic Adsorption by BCXZM Composite

**Figure 2:**
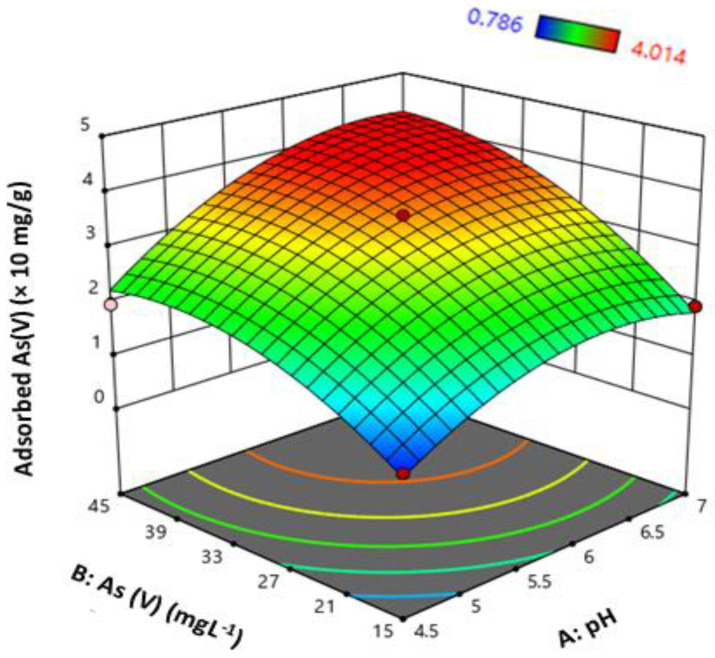
Response surface for (CCD)2^2^ experiment of Adsorbed As(V) by BCXZM composite.

pH has a very significant role in arsenic removal as the overall charge on a biosorbent as well as the ionic nature of the As is strongly dependent on it [22]. In the current study, the effect of pH on SBB of As(V) was studied at a range of 3.9 to 7.5. The results of the effect of arsenic concentration and pH on arsenic adsorption are shown in Figure 2. The (CCD)2^2^ design revealed that arsenic adsorption was strongly affected by both pH and As(V) concentration. It can be seen in Table 2 that both are significant factors in quadratic model applicability, with F-values of 78.80 (*p* ≤ 0.0003) and 127.20 (*p* < 0.0001) for pH and As(V) concentration, respectively. The F-value for a model lack of fit is 15.64 (*p* ≤ 0.05), which is less than 53.48 (model significant F-value), which implies that the model fitting is accurate due to the experimental factors [35]. Once the pH was <4.0, the bio-removal of As(V) was very low (0.86 to 1.96 × 10 mg/g). With the rise in pH from 5 to 7.0, there was a radical rise in the adsorption of As. At pH 7, the highest adsorption capacity was attained for 45 mg/L As(V), while at pH < 5, the arsenic adsorption was significantly (*p* ≤ 0.0003) reduced at the same As(V) levels. Studies in the past have also confirmed the successful application of such models for the optimization of arsenic biosorption process [8,27,28,36].

This is a dually influential process: the removal of As(V) by BCXZM is affected by the surface charges of the composites as well as the ionic form of arsenic that is altered with the pH fluctuations [37]. Previous studies have confirmed that As(V) is mainly in the form of H_2_AsO_4_^−^ when the pH of the solution is between 3 and 6, while with a pH between 6 and 8, arsenic is present as both monovalent (H_2_AsO_4_^−^) and divalent anions (HAsO_4_^2−^) [38]. One more aspect of the reduced pH is the protonation of the bacterial cell wall [39] due to a high amount of H^+^ ions at acidic pH, while at a pH between 5 and 6, de-protonation of reactive sites occurs and mostly phosphoric, amino groups, and carboxylic, groups bind As(V) to bacterial EPS [33,40].

At an acidic pH, the cell walls of the bacterial cells were much protonated, which might have resulted in arsenic reduction. The rise in pH from 5 to 7 resulted in de-protonation and increased bacterial growth, which are responsible for the strong electrostatic interaction between positively charged BCXZM surface, oxyanions and arsenic. The dominant species of As(V) in the above-mentioned pH range are H_2_AsO_4_^−^ ions, which can be adsorbed on the BCXZM composite by replacing hydroxyl ions or hydroxyl groups coordination [39]. The reduction in the As(V) adsorption at a pH > 7 may be justified to the negative charge (due to excess hydroxyl ions) on the bacterial cell wall in the BCXZM composite and the decrease in EPS production (Figure 1b) in these conditions. Therefore, the repulsion between a negatively charged cell wall and arsenic anions and reduced EPS production might have resulted the reduced As(V) adsorption by the BCXZM composite [14,24].

The strain XZM also produces EPS that increases with the increasing As(V) concentration (Figure 1a) and is maximum at pH 7 (Figure 1b). The EPS acted as a key material for the attachment of the As(V), thus assisting the bacteria to grow at high concentrations of arsenic [24,33,41], and ultimately promoted arsenic adsorption in the SBB process. The fluctuations in the surface properties have proven to be one of the major factors affecting the adsorption of arsenic [19,21,38].

### 3.3. Optimization and Quadratic Model Equation for Maximum Arsenic Adsorption

The RSM/(CCD)2^2^ model was used to formulate a polynomial of fitted quadratic model that explains the relationship between experimental factors (pH and As(V) concentration) and response (q). Equation (3) was simulated using the (CCD)2^2^ model that was used to determine the maximum arsenic adsorption value by the BCXZM composite.

(3) $$q=-15.62621+4.94099\times pH+0.147783\times As\left(V\right)+\;0.013147\times pH\times As\left(V\right)-0.411093\;pH^{2}+0.00265\times As{\left(V\right)}^{2}\;$$

From the above model simulation, the maximum As(V) adsorption capacity of BCXZM of 4.23 (×10 mg/g) was predicted at a pH 6.9 and concentration of 48.9 mg/L. The experiment was performed in these suggested optimized conditions and arsenic adsorption of 4.26 (×10 mg/g) was achieved. Recently, a study on the SBB of arsenic [42] has also presented the similar results. This similarity further encouraged the application of the (CCD)2^2^ model for the optimization of As bio-removal by SBB.

### 3.4. SEM of Bacterial Growth Changes in BCXZM Composite

**Figure 3:**
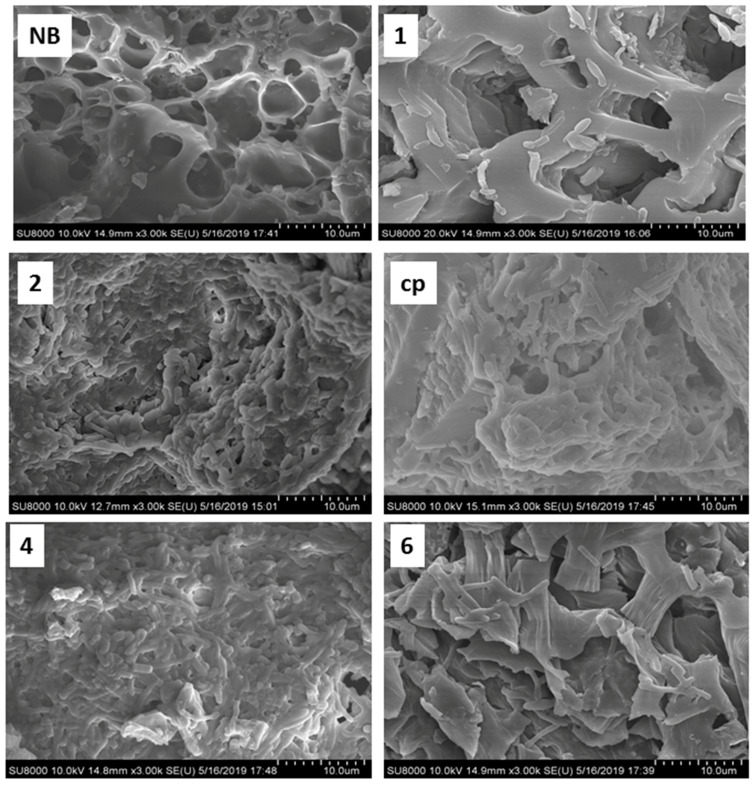
SEM micrographs of the bacterial biofilm formation on multifunction biochar at different pH values and arsenic concentrations. Note: NB: native biochar, 1, 2, 4, 6 and cp: correspond to the experimental conditions according to Table 1.

The SEM micrographs of the biochar with and without *Bacillus* XZM immobilization are shown in Figure 3. This figure also depicts the fluctuations in the bacterial growth and EPS production at different pH values after As(V) adsorption. The porous structure of the biochar without bacteria is very much visible in sample NB, while in sample 1, at pH 4.5, very little bacterial growth has occurred and no EPS matrix is visible. In contrast, in samples 2 and 4, at pH 7, a large amount of bacterial colonies can be observed immersed in the thick EPS matrix. The high arsenic adsorption at this pH can be attributed to this EPS [24], which is further confirmed by the highest amount of EPS production by *Bacillus* XZM at pH 7 (Figure 1b) and FTIR peak shuffles in hydrocarbon groups. The partially deformed bacterial cell structures in sample cp and sample 6, at pH 5.5 and 7.5, correspond to the reduced *Bacillus* XZM growth at this pH range [34]. As a result, reduced arsenic adsorption is achieved in these samples (Figure 2). Overall, the reduced bacterial growth at acidic pH and EPS production, as well as maximum *Bacillus* XZM growth on BCXZM composite at pH ~7, are verified through the SEM studies.

### 3.5. EDX Spectroscopy for Maximum Arsenic Adsorption

**Figure 4:**
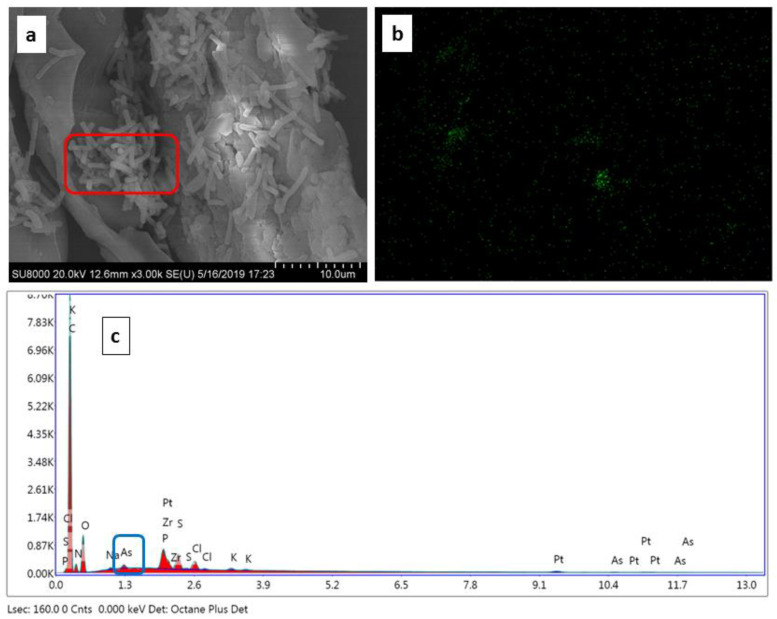
Arsenic accumulation and bacterial immobilization on BCXZM composite in optimized experiment. (**a**) SEM micrograph; (**b**) elementary overly of arsenic; and (**c**) EDX graph of the BCXZM composite in optimized experiment.

**Figure 5:**
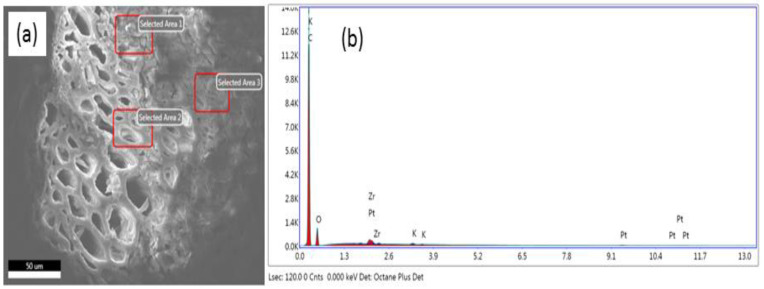
(**a**) SEM micrograph of biochar and (**b**) EDX graph of biochar after arsenic adsorption at optimized conditions.

In order to further reinforce the arsenic adsorption by BCXZM the SEM micrograph, elemental overlay of arsenic and EDX graph of the BCXZM composite after arsenic adsorption in optimized condition (Section 3.3) are given in Figure 4a, 4b and 4c, respectively. The arsenic peak appeared at approximately 0.6 K represents arsenic adsorption by BCXZM composite [43]. The elemental overlay (Figure 4b), as green intensities, represents the arsenic distribution in SEM micrograph (Figure 4a). It is clear from the elemental overlay that there is more arsenic adsorption on BCXZM at the spots with high bacterial growth/colonies than in the less/no growth points. For the validation of this point, the SEM micrograph and EDX graph of the native biochar after arsenic adsorption (under same conditions) are presented in Figure 5a and 5b, respectively. The absence (too low to be detected) of arsenic peaks in this micrograph is an indication of the fact that, in these optimized conditions, the BCXZM can adsorb more arsenic than simple biochar. These results provide further confirmation of the role of *Bacillus* XZM in the enhanced arsenic adsorption by BCXZM. It is inferred that the *Bacillus* XZM plays a major role in arsenic transformations and enhances the surface properties of biochar for adsorbing more arsenic. These contributions of bacteria make the SBB of As(V) by BCXZM composite a very productive process compared to simple biosorption by biochar. The current study is the first to report the role of bacteria in the SBB process.

### 3.6. FTIR Spectra of BCXZM Composite after Arsenic Adsorption

**Figure 6:**
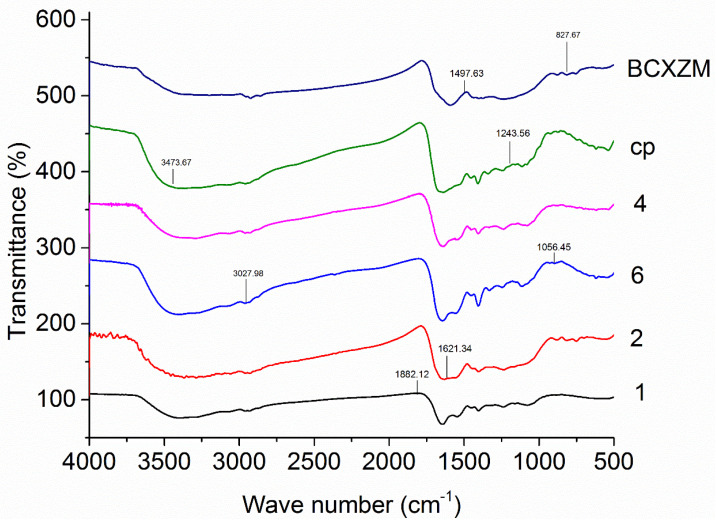
FTIR spectra of the native biochar and BCXZM composites after As(V) adsorption. Note: 1, 2, 4, 6 and cp correspond to the experimental conditions according to Table 1.

The adsorption of As(V) on the BCXZM composite is strongly dependent on its surface functional groups. The FTIR spectra of the BCXZM composite before and after adsorption of As(V) (in trail 1, 2, 6, 4 and central point) are presented in Figure 6. After adsorption of arsenic, at different pH values, a change in the FTIR spectra of the BCXZM composite was recorded at 827.67, 1056.45, 1243.56, 1497.63, 1621.34, 1882.12, 3027.98 and 3473.67 cm^−1^. The peak shifts at 3027.98 and 3473.67 cm^−1^ might be attributed to the change in the –NH and –OH groups with the pH fluctuations [44]. These results are also consistent with the increased and reduced arsenic adsorption at pH 7 and <5, respectively. The stretching at 188.12 cm^−1^ belongs to the aliphatic hydrocarbon (–CH). The *Bacillus* XZM growth and EPS production is affected by pH, which ultimately affects the hydrocarbons contents [21] on the surface of the BCXZM composite. These aliphatic bands are also responsible for the adsorption of As(V), and the increase in these bands at pH 7 might also have increased the arsenic adsorption. The peak shifts at around 1699.44 and 1745.38 cm^−1^ are ascribed to the –C=O stretching in the form of aliphatic acids [31]. A very intense band shift appeared at 1621.34 and 1497.63 cm^−1^, which might be attributed to the aromatic/-NO_2_ group. These peaks have also been observed for the adsorption of As(III) in the SBB process [19,42,43]; here, recognition of the arsenic redox activity of the bacteria is important. The bacterial As transformation converts it into different forms. Some of these chemical forms of arsenic can be: arsenate As V (AsO_4_^3−^ HAsO_4_^2−^, and H_2_AsO_4_^1−^), arsenite AsIII (As(OH)_3_, As(OH)^4−^, AsO_2_OH^2−^, and AsO_3_^3−^), methyl-arsonic acid CH_3_AsO(OH)_2_ (MMA) and dimethyl-arsinic acid (CH_3_)_2_AsOOH (DMA). This may also facilitate the As adsorption on the BCXZM composite providing a wide range of As compounds that can be attached to a number of functional groups, according to the reactivity parameters [5,11,24], rather than just one specific chemical form that may avail limited attachment sites (as in simple biosorption processes). The peak shuffle at 1243.56 cm^−1^ can be ascribed to the sulphonyl group, which has also been previously observed in the SBB of As [25]. The peak at 1056.124 cm^−1^ may be due to the –C–N stretching, particularly of amino groups, that can be attributed to the interaction of nitrogen from the amino group with arsenic [30]. The small peaks at 827.67 cm^−1^, disappearing with the decreasing pH (from BCXZM to sample 1), might be attributed to -COO bands that are essentially polysaccharides [45].

### 3.7. Desorption Studies of Arsenic from BCXZM Composite

**Figure 7:**
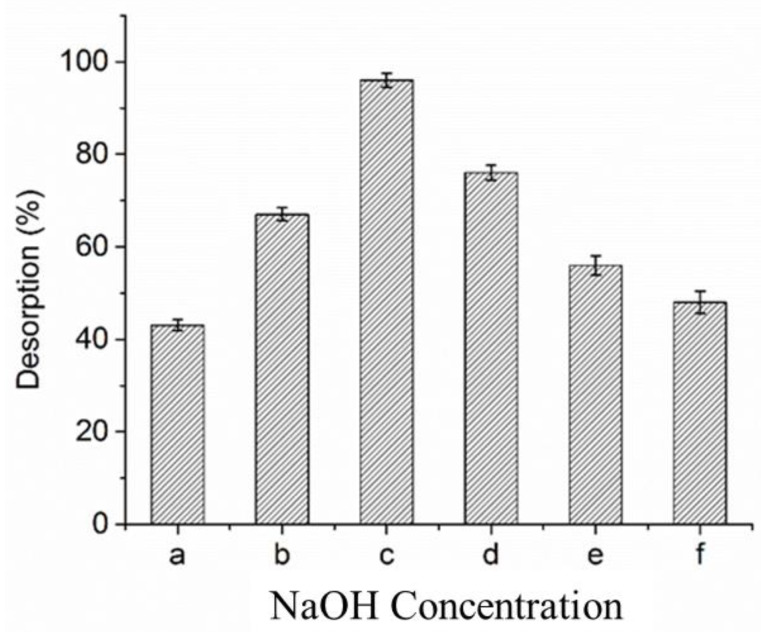
%Desorption of arsenic from BCXZM composite after optimized experiment. Note: (a) 0 M, (b) 0.01 M, (c) 0.05 M, (d) 0.5 (e) 0.1 M and (f) 1 M NaOH; Mean ± Standard deviation (n = 2).

In the SBB process, arsenic is bioaccumulated/biosorbed on the BCXZM composite; once the maximum adsorption capacity of arsenic (4.23 × 10 mg/g, Figure 2, Equation (3) and Section 3.3) is reached, the lag phase of the bacterial growth may start and/or all the available sites of the functional groups may be occupied by arsenic. Therefore, the desorption study becomes a necessary part for the practical applicability of SBB process, as it is important to know the percentage recovery of biochar so that it can be reused [23] in the bacterial immobilization surface, especially once applied in bioreactors. The maximum rate of arsenic desorption (~87%) was observed at 0.01 M NaOH level; further rises in the NaOH level reduced the arsenic desorption (Figure 7). The reduction in the desorption of arsenic has also been observed in previous studies on SBB; however, maximum desorption was found at 0.05 M NaOH. This might be attributed to the properties of *Bacillus* XZM; as an individual species, each bacterium has different attributes [11,41]. Desorption of arsenic at alkaline pH might be attributed to the de-protonation of the –OH group at high pH, which might have resulted in a negative charge on the surface of BCXZM. As a result of this, there were dominant repulsive forces between arsenic oxyanions and negatively charged BCXZM surface leading to the desorption of arsenic [7,19,31]. Therefore, once the maximum adsorption capacity of the BCXZM composite is reached, the biochar can be reused after desorption as supporting material to grow biofilm, and newly prepared BCXZM can be used for arsenic bioremediation. As BCXZM biofilm can work effectively at a high As(V) concentration of 45 mg/L (Table 1), it is recommended, for future studies, to use bio-filters based on BCXZM for the arsenic bioremediation of drinking water. The construction and techno-economic analysis of the bio-filters based on BCXZM composite is a future prospect of our arsenic bioremediation study.

### 3.8. Cost of BCXZM Preparation

At the maximum adsorption capacity of 42.3 mg/g, 23 g of BCXZM is required for 1 g of As(V) adsorption. In our experiment, 0.4 g of oven-dried biochar resulted in approximately 2 g of BCXZM composite (Section 2.3 and Section 2.5). This can be explained by the water absorption capacity of biochar and mass of biofilm; depending upon its origin, biochar can absorb water up to 5 times its weight [46]. Therefore, approximately 5 g of biochar is required in 7.608 L of water to make 23 g of the BCXZM composite. Considering the electricity and nutrient costs, USD 32.99 were required to prepare the BCXZM composite to remove 1 g of As(V). If we consider that drinking water has an arsenic concentration of 50 µg/L, it will cost 6.244/1000 gallons of water (Table 3). This value is equal to/less than those of the existing methods of arsenic remediation used on commercial scale. Moreover, this is an estimate for a very small scale; once the scale increases, the per unit treatment cost will decrease further [47].

The application of the RSM/CCD is suggested for the optimization of the SBB process, which can further provide a basis for designing and operating bioreactors for arsenic remediation in future. This study is also the first to report the determination of maximum adsorption As(V) capacity in the SBB process through RSM/(CCD)2^2^ application. This study also highlighted the individual role of the bacteria in the SBB process in terms of EPS production, which has been overlooked by previous studies. It is also inferred that the SBB process is very different from simple biosorption processes; therefore, it is more practical to optimize this process with a less laborious RSM or other multivariate optimization models rather than perceiving it as a simple biosorption process or determining its possible fittings in isothermal models. Recently, a comparison between chemomertic and artificial neural network (ANN) techniques has been made in the field of arsenic adsorption [48], and further application of ANN for the optimization of SBB process is a key future prospect of the current study. This research is a step for future studies on the optimization of the SBB process with various parameters (e.g., temperature, flow rate, retention time, and dosage, etc.); batch as well as column processes are required for more interpretations. Once detailed information of all these parameters is achieved, the low-cost BCXZM composite can be used in bio-based water filters. The molecular composition of EPS produced by *Bacillus* XZM at different pH values under arsenic stress is also a limitation of the current study that may prove more helpful in revealing the more detailed insights of the SBB process.

## 4. Conclusions

The maximum arsenic adsorption capacity of 4.23 × 10 mg/g is predicted and achieved by (CCD)2^2^ for the BCXZM composite in the current experimental conditions, which was also verified using SEM-EDX graph and elemental overlay. The arsenic adsorption by the BCXZM composite is higher than that of native biochar alone in the same conditions. *Bacillus* XZM played an important role in the adsorption of arsenic through EPS production. Bacterial EPS production was found to be maximum at pH 7, and reduced considerably at acidic and basic pH ranges. Due to this pH sensitivity of the *Bacillus* XZM EPS, –NH, –OH, -CH, –C=O, –C–N, –SH, –COO and aromatic/-NO_2_ functional groups, on the surface of BCXZM, were responsible for arsenic adsorption. Furthermore, the *Bacillus* XZM was found to be responsible for the transformation of As(V) to the many other chemical forms, such as arsenite AsIII (As(OH)_3_, As(OH)^4−^, AsO_2_OH^2−^, and AsO_3_^3−^), methyl-arsonic acid CH_3_AsO(OH)_2_ (MMA) and dimethyl-arsinic acid (CH_3_)_2_AsOOH (DMA), resulting in an enhanced adsorption of arsenic by the BCXZM composite rather than simple biochar. This study has also reported the role of bacterial arsenic transformations and EPS production in maximum arsenic adsorption capacity of a biosorbent–bacteria composite. In addition to this, approximately USD 6 are required to prepare the BCXZM composite for 1000 gallons of drinking water with an As(V) level of 50 µg/L. This cost further confirms the feasibility of the construction of a biological drinking water filter based on BCXZM in the future.

## Figures and Tables

**Table 1:** Results of the (CCD)2^2^ experiment in terms of arsenic adsorbed by the BCXZM composite.

Experiment	pH	As(V) (mg·L^−1^)	q (×10 mg/g)
1	4.5	15.00	0.86 ± 1.23
2	7.00	15.00	1.93 ± 1.16
3	4.50	45.00	1.96 ± 0.97
4	7.00	45.00	4.01 ± 1.31
5	3.98	30.00	1.25 ± 0.78
6	7.51	30.00	3.34 ± 0.85
7	5.75	8.78	0.78 ± 1.58
8	5.75	51.21	3.99 ± 1.02
9 (cp *)	5.75	30.00	3.46 ± 1.30
10 (cp)	5.75	30.00	3.59 ± 0.99
11 (cp)	5.75	30.00	3.46 ± 1.08

* Central Points, Mean ± SD.

**Table 2:** ANOVA for the quadratic model fitted for the As(V) adsorption by BCXZM composite.

Source	Sum of Squares	df	Mean Square	F-Value	*p*-Value *	
Model	15.6	5	3.13	53.48	0.0002	Significant
A-pH	4.62	1	4.62	78.80	0.0003	
B-As(V)	7.45	1	7.45	127.20	<0.0001	
AB	0.24	1	0.24	4.15	0.0673	
A^2^	2.33	1	2.33	39.76	0.0015	
B^2^	2.01	1	2.01	34.27	0.0021	
Residual	0.29	5	0.058			
Lack of Fit	0.281	3	0.0937	15.64	0.0507	not significant
Pure Error	0.012	2	0.006			
Cor Total	15.9	10				

* *p*-Value ≤ 0.05 represents significant at 95% confidence interval.

**Table 3:** Cost of BCXZM preparation for the adsorption of 1 g As(V), based on the adsorption capacity of 42.3 mg/g.

	Amount Required	Cost (USD)
Biochar	5.75 g	0.05
Bacterial biofilm formation (electricity kw)	7.608	0.699
Peptone	35.9375 g	17.32
Beef extract	14.375 g	6.21
NaCl	35.935 g	5.66
Deionized water for biofilm preparation	7.187 L	2.355
Electricity for arsenic removal (kw)	7.608	0.699
Total cost of BCXZM preparation for 1 g of As(V) adsorption		32.993
If the As concentration in drinking water is considered as 50 µg/L [47]		
Volume of water with 1 g of arsenic (Gallons)	5283.441	
BCXZM cost for 5283.441 gallons (USD)	32.993	
BCXZM cost per gallon (USD)	0.006244	
BCXZM cost per 1000 gallon (USD)	6.24460	

## Data Availability

Not applicable.

## Supplementary Materials

The following supporting information can be downloaded at: https://www.mdpi.com/article/10.3390/biology12040611/s1, Figure S1: EPS formation by *Bacillus* XZM at different Temperature; Figure S2: Removal efficiency of the As(V) at different (a) dose of BCXZM composite and (b) time interval while keeping dose of BCXZM at 2 g/L; Table S1: Factors and levels for the (CCD)2^2^ experiment of arsenic adsorption by BCXZM composite.
