# Supplementary material for: Application of BCXZM Composite for Arsenic Removal: EPS Production, Biotransformation and Immobilization of Bacillus XZM on Corn Cobs Biochar
Source: Biology (Basel). 2023 Apr 18;12(4):611. doi: 10.3390/biology12040611 (PMC10136123; doi:10.3390/biology12040611)
Supplement: Supplementary file 1 [file biology-12-00611-s001.zip › biology-2292854-supplementary.pdf]

Table S1: Factors and levels for the (CCD)$2^2$ experiment of arsenic adsorption by BCXZM composite.

| Factor | Name | Low Level | Middle Level | High Level |
|---|---|---|---|---|
| A | pH | 4.5 | 5.75 | 7 |
| B | As(V) | 15 | 30 | 45 |

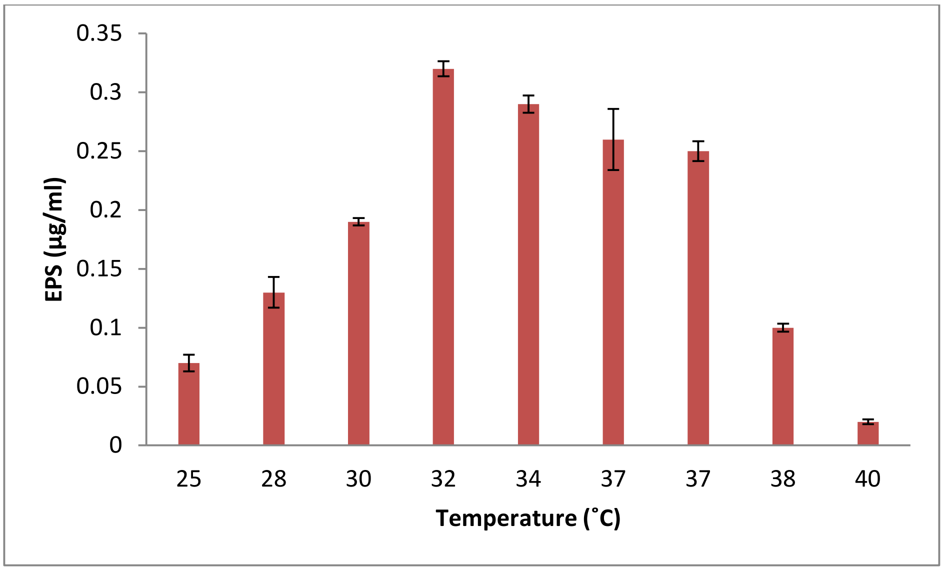


Figure S1: EPS formation by *Bacillus* XZM at different Temperature

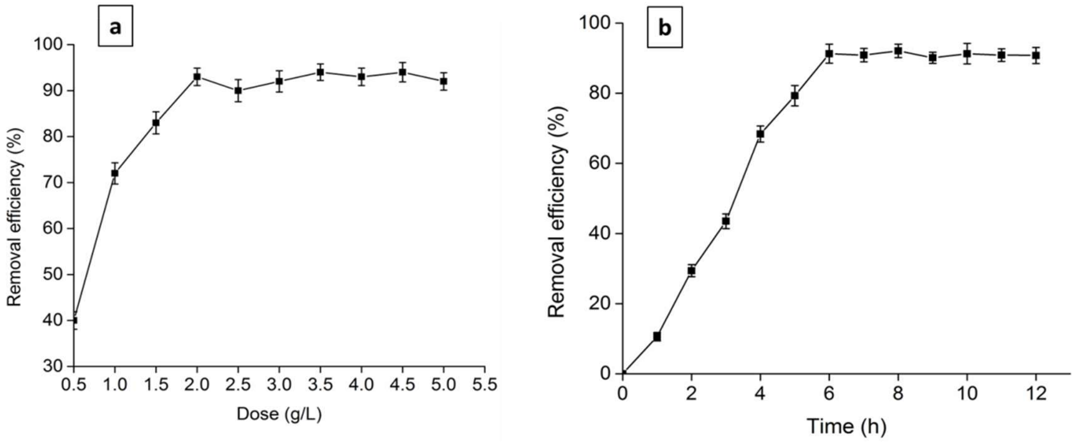


Figure S2: Removal efficiency of the As(V) at different a) dose of BCXZM composite and b) time interval while keeping dose of BCXZM at 2 g/L
